# Pandemics–Keep Calm and Carry On

**DOI:** 10.1371/journal.pbio.1001780

**Published:** 2014-02-04

**Authors:** Edward C. Holmes

**Affiliations:** Marie Bashir Institute for Infectious Diseases and Biosecurity, School of Biological Sciences and Sydney Medical School, The University of Sydney, Sydney, Australia

## Abstract

Evolutionary biologist Edward Holmes reviews Peter Doherty's book, *Pandemics: What Everyone Needs to Know.*

**Figure:**
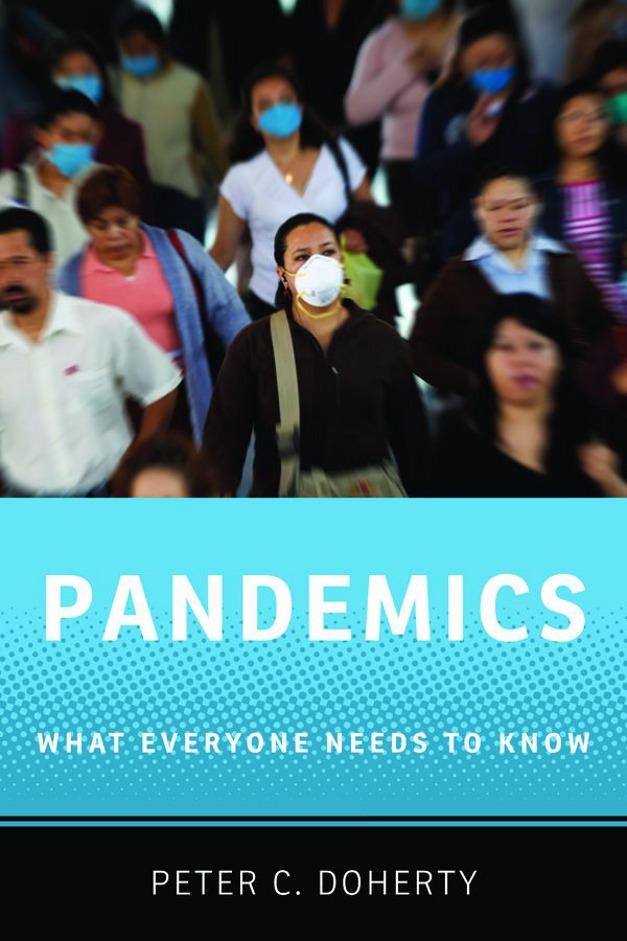
Doherty PC (2013) Pandemics: What Everyone Needs to Know. New York: Oxford University Press. 222 pp. ISBN 978-0-19-989812-1 (paperback).

How best to prevent and control an infectious disease pandemic is one of the major challenges facing modern biomedicine, with inputs and impacts beyond the purely scientific. The potential for novel microbial pathogens to emerge and cause mass sickness and death has inspired often frightening and sometimes fantastic tales, with the distinction between fact and fiction often blurry at best. Globalization has aided the rapid spread of ancient and novel pathogens at a time when the drugs to combat them are losing their effectiveness as the pathogens evolve resistance. Without a reasoned view it might be easy to believe that the human species is a face mask away from extinction. A factual, yet accessible, account of how pandemics arise and what we might do to prevent them would therefore be a valuable asset. Peter Doherty's new book, *Pandemics: What Everyone Needs to Know*, provides a calm and authoritative counterpoint to pandemic scare-mongering, and manages to entertain at the same time.

*Pandemics: What Everyone Needs to Know* is written as a series of question-and-answers on a diverse array of topics that relate to pandemics, as might be asked by an interested member of the general public or a specialist seeking broader knowledge. Groups of related questions form the basis of individual chapters. The book finishes with a useful bullet-point summary of the main words of advice, which can almost stand alone as a quick-glance reference guide or course synopsis for “Pandemics 101.”

If knowledge is power then this book is truly liberating. The text is impressive from a variety of angles; the breadth of topics covered, which range from proteins to politics, the sure-footed and fact-laden response to each, and the highly accessible style. It is a cliché to say that an author is uniquely qualified to write a particular book, but Doherty's five decades of related research, which resulted in a Nobel Prize in Physiology or Medicine (with Rolf Zinkernagel) in 1996, as well as roles on advisory panels, mean that in this case the cliché is justified. The book is enjoyable for its lively turn of phrase and Aussie directness, and shows that the author is someone who clearly does not mind getting his hands dirty. Doherty's writing has an intimacy that feels like a fire-side lecture from a knowledgeable and somewhat glamorous uncle, and I found myself noting lots of memorable phrases to be stored for future use. For example, when discussing respiratory diseases Doherty asks the question “What is snot?” The answer, by the way, is “Basically, snot and phlegm are mucus plus junk.”

As expected given his background, Doherty devotes a fair amount of space to two of his main research interests—immunology and influenza—and the book starts with a useful nuts-and-bolts look at infection and immunity. However, this is in no way an exercise in self-indulgence; rather, it is an attempt to inform on biology and policy that relates to pandemics, with education the key goal. In doing so, Doherty provides a brief, but potent, update on state-of-the art science and does not shy from complexity where necessary. For example, he provides an excellent account of why influenza's rapid respiratory mode of transmission, combined with often high virulence and an abundant animal reservoir, still make it the pathogen of choice for a devastating human pandemic. However, he never raises the temperature unnecessarily and reasoned and carefully chosen words are deployed throughout. Indeed, it is fair to say that Doherty is cautiously optimistic about our prospects of dealing with the next “big one,” with continued scientific advances, and our increasingly connected disease response system, a reason for hope. The text is also littered with informative side anecdotes and scientific history; good behind-the-scenes stuff that provides a fascinating back-story to many of the things we take for granted, such as the origin of the influenza strains that have become laboratory staples, and even the meanings of childhood rhymes (some of which, unsurprisingly, involve snot). My only negative comment is that the small number of figures are rather perfunctory, of poor quality, and not fitting the high quality of the text. A little investment in color would have gone a long way.

The power of this book is its scope, with pandemics explored from every possible angle, so much so that in parts it becomes a more general reflection on the human condition. Hence, as well as biology, *Pandemics: What Everyone Needs to Know* contains excellent public health advice, such as when to get HIV tested and the value of face masks. There are also sections on the workings of government, the pros and cons of the current pandemic classification system, and the threat of a pandemic due to bioterrorism or a rogue state, building on the highly controversial gain-of-function experiments in H5N1 influenza virus [1],[2]. Doherty is also right up-to-date on the role that social media can play in the early detection of and response to pandemics, which will surely grow in importance [3]. One of the most useful sections includes advice on how each individual can protect themselves during a pandemic, which can be roughly summarized as (a) be aware and alert, (b) calmly follow the informed media, (c) make use of advice hotlines, and (d) follow the correct coughing etiquette (i.e., avoid hands as much as possible). Doherty's Australian heritage is reflected in the well-told story of how the deliberate releases of myxomatosis and rabbit hemorrhagic disease as agents of rabbit biocontrol provide unique information on the patterns and dynamics of virgin-soil epidemics, and hence are a powerful analogy to natural disease emergence [4]. He also advocates a reasoned response to the plethora of viruses now being discovered in bats and rodents, to which we might be occasionally exposed [5]—“Avoiding infection with any diseases that such species carry is simply a matter of being aware and, when necessary, modifying our own behavior.”

Effective pandemic prevention and control requires a social contract in which we trust government, itself informed by good science, to act promptly and effectively on our behalf, even in the face of ill-informed public opinion. It is therefore apt that Doherty ends by noting the essential unity of human society when faced by a pandemic: “Infectious diseases are no respecters of wealth, power, or personal merit. Pandemic infectious disease is one situation where we cannot accept Margaret Thatcher's view that ‘there is no such thing as society’. With a fast-spreading respiratory virus, for example, everyone is ultimately all in the same boat.” Rather than filed away on a dusty shelf, this book merits being keep next to your medicine cabinet.

**About the Author**

Dr. Edward C. Holmes is an NHMRC Australia Fellow and Professor at the University of Sydney, Australia. He completed his Ph.D. at the University of Cambridge, and has held faculty positions at the University of Oxford and the Pennsylvania State University before moving to Sydney in 2012. His research focuses on the evolutionary genetics of viruses, the molecular epidemiology of human and animal pathogens, and the evolutionary processes that underpin viral emergence.
